# Personalized Nutrition Advice: Preferred Ways of Receiving Information Related to Psychological Characteristics

**DOI:** 10.3389/fpsyg.2021.575465

**Published:** 2021-06-22

**Authors:** Garmt B. Dijksterhuis, Emily P. Bouwman, Danny Taufik

**Affiliations:** ^1^Wageningen Food & Biobased Research, Wageningen University & Research, Wageningen, Netherlands; ^2^Wageningen Economic Research, Wageningen University & Research, Wageningen, Netherlands

**Keywords:** health advice, nutrition advice, psychological scales, personalized nutrition and health advice, psychosocial characteristics

## Abstract

The primary goal of this study is to be able to discern specific types of consumers in terms of their psychosocial characteristics who may need different ways of receiving dietary advice. Knowing these types will enable a better fit of advice to consumers’ psychosocial characteristics, hereby stimulating healthy eating as the probability of compliance to the advice can potentially increase. The study draws upon several psychological theories to distinguish unique underlying factors that can subsequently be used to personalize nutrition information for consumers. A number of general psychological scales (self-regulation, action and coping self-efficacy, social comparison, intrinsic motivation, health info processing, need for cognition and for affect, and regulatory focus) are filled out by 988 respondents, including their preferences for receiving personalized forms of nutrition advice. The set of joint items from various psychological constructs is analyzed using a Principal Component Analysis to find underlying psychological characteristics. The PCA produces four components (explaining 51% of variation), that could be interpreted as ‘intrinsic interest and capabilities for healthy eating,’ ‘perceived difficulty to eat healthily,’ ‘self-worth insecurity,’ and ‘seeking positive challenges,’ respectively. By means of a Logistic Regression these components are able to predict preferences for different forms of receiving nutrition advice. This first component shows that a mind set for maintaining a healthy diet goes together with an interest in receiving an advice on what do to and on how that will affect one’s health. The second component predicts a preference for a fixed moment to receive information/advice. This may be a strategy of those that perceive difficulties to eat healthily, to help them control their healthy food intake. The insecurity that the third component models seems to lead to a wish for receiving specific advice about their health situation at fixed moments in time. The fourth component is a small component, therefore its prediction of a wish for an advice focussing on prevention of negative consequences is probably not a strong result. The study does point out that there appear different psychosocial types of consumers, that may benefit by being addressed according to their preference for receiving nutrition advice on specific moments, of a specific level of detail or pointing at the type of consequences the advice has. A better fit of the advice to the psychosocial characteristics of the recipient, captured in the identified components in the current study, may lead to an increase in compliance, although that will have to be further investigated in subsequent work.

## Introduction

Many people in the Western world currently have an unhealthy lifestyle, which in part is the result of consumers’ diets which overall are relatively unhealthy. For instance because they consume too much meat, have a high (saturated) fat intake, and consume too little fruits and vegetables (Bray and Popkin, 1998; Pomerleau et al., 2005). Such unhealthy diets increase the odds of consumers getting various (chronic) diseases, such as diabetes or cardiovascular diseases (Wing et al., 2001). One type of approach to persuade consumers to eat more healthily is to provide consumers with nutrition information (e.g., Block and Peracchio, 2006; Glanz et al., 2012; Wendel et al., 2013). This nutrition information can be provided to consumers in a generic manner, where all consumers receive the same information (in terms of content and form), or in a more personalized manner. The concept of personalized nutrition in this study entails that nutrition information is provided to consumers taking into account some of their psychological characteristics. Consequently, content and/or form of the information can be personalized based on these characteristics. The aim is that this will make the provided nutrition information more personally relevant (e.g., Brug et al., 1999), in turn leading to a higher compliance. Perceiving more benefits than risks drives this higher compliance and ambivalent feelings and eating context can be barriers (Berezowska et al., 2015; Reinders et al., 2020). Overall, personalizing nutrition information has been shown to be more effective in affecting consumers’ food choices than providing consumers with generic nutrition information (e.g., Brug et al., 2003; Elder et al., 2009; Livingstone et al., 2016; Celis-Morales et al., 2017), which also demonstrates the potential of personalized nutrition advice as a strategy to ultimately reduce the incidence of diseases and consequently health costs (Stewart-Knox et al., 2016).

Nutrition information can be personalized in different ways. A distinction can be made between a biological/medical basis on the one hand and a behavioral/psychological basis on the other hand (Ordovas et al., 2018). The majority of studies that have addressed effects of personalized nutrition on consumers’ food choices have used a biological/medical basis to personalize nutrition information. Examples are a personalized nutrition approach based on blood parameters, anthropometrics and dietary habits (Zeevi et al., 2015), on epigenetics (vel Szic et al., 2015) or on nutrigenomics (Ronteltap et al., 2013; Berezowska et al., 2015).

Behavioral or psychological approaches to personalize nutrition information have been more scarce. Many behavior change techniques used in this field originate from psychological theories (Peters et al., 2015). Psychological characteristics of consumers can strongly affect consumers’ behavior, such as the food choices they make (e.g., Mela, 2001; Gibson, 2006; Köster, 2009), although to our knowledge these psychological insights have not yet been applied to personalize nutrition information. Macready et al. (2018) developed a study protocol for a personalized nutrition approach based on multiple behavior change techniques, which included the Theory of Planned Behavior (Ajzen and Manstead, 2007), social cognitive theory (Bandura, 1989), and the Information-Motivation-Behavioral Skills Model (Fisher et al., 2002). However, identifying which behavior change techniques, based on psychological theories, are most relevant to include in a personalized nutrition intervention is challenging, as the practice of including these is currently still mostly exploratory (Macready et al., 2018).

While each theory will have its own merits, there is overlap between the psychological theories in the sense that they draw on either individual cognitions, capabilities and motivation, or on contextual and/or social variables (Davis et al., 2015). This overlap makes it difficult to pinpoint which factors can be distinguished, as also noted by Macready et al. (2018). The primary goal of this study is to draw upon several psychological theories in an attempt to distinguish unique underlying factors that can subsequently be used to personalize nutrition information for consumers, to ultimately stimulate healthy eating. A secondary goal is to explore the relation between the identified factors and consumers’ preferences for receiving certain forms of dietary advice/information. In this way, we check to what extent the identified factors can provide a clearer and hopefully leaner psychological basis for personalizing nutrition information.

A meta-analysis of Noar et al. (2007) points at several behavior change techniques and psychological characteristics that can be effective for personalized approaches to promote healthy behavior in general (not specifically in relation to nutrition). Their overview, combined with the behavior change techniques and underlying psychological characteristics as used by Macready et al. (2018), provide our starting point to make a selection of psychological theories. Below we provide a short overview of the theories we draw upon, including a short elaboration on why they could be useful for personalizing nutrition information.

We stress that our approach is of an exploratory nature, and not hypothesis-driven. We include a set of scales based on them potentially capturing relevant psychological traits, not based on theoretical considerations about what traits may cause a specific type of consumer to prefer specific types of food and health related information.

### Psychological Theories as a Basis for Personalizing Nutrition Information

Personalized nutrition advice is a tool to help individuals regulate their dietary behaviors. In the current study, we take into account various psychological theories focused on individual differences, in terms of capabilities and dispositions that potentially affect preferences for dietary advice and whether the advice will be processed and in turn can affect compliance rates and dietary behaviors. Following an advice mainly involves conscious processes, like goal setting and self-monitoring (Rankin et al., 2017). Social Cognitive Theory (SCT; Bandura, 1977) gives insight into how individuals regulate their behavior to achieve goals that can be maintained over time. Bandura (1991) elaborates that an interaction between personal factors (self-reflective capabilities that give individuals some control over their thoughts, feelings, motivation and actions) and environmental matters (e.g., social influences, role models, behavioral standards) influences self-regulation skills and behavior. Therefore, we included *self-regulation*, *self-efficacy* (the extent to which one feels capable of performing a certain behavior) and *social comparison* in our study. Within the Health Action Process Approach model (HAPA; Schwarzer, 1992, 1999) *self-efficacy* is further subdivided into *action self-efficacy* and *coping self-efficacy*. *Action self-efficacy* refers to the capability to imagine success scenarios, anticipate potential outcomes of strategies and initiate new behaviors. *Coping self-efficacy* refers to the capability to deal with barriers that arise. Both *action self-efficacy* and *coping self-efficacy* are included in our study. Based on Self-Determination Theory, a motivational theory that gives insight into the reason behind self-regulation (SDT; Ryan and Deci, 2000), we also included *intrinsic motivation*. *Intrinsic motivation* is referred to when one is enjoying an activity in itself, without other reasons than performing the activity. Intrinsic motivation has been shown an important predictor of long term behavior change (Ryan and Deci, 2000; Orji et al., 2013).

Personalized advice can increase the effect of a message by achieving a fit between a person and a message. According to the Elaboration Likelihood Model (ELM; Petty and Cacioppo, 1986), information can be processed in two different ways, namely via the content of a message, i.e., based on its meaning (the central processing route) or via the appearance of a message (the peripheral processing route). The central route is used when individuals are motivated, capable and able to attentively take in information. When this is not the case, the peripheral route is used. When it comes to healthy diets, there are differences between individuals, in motivation, capability and ability (e.g., Dibsdall et al., 2003), which according to ELM leads to different information processing routes (Cacioppo et al., 1984; Trumbo and McComas, 2003). Therefore, we included *information processing* from ELM (central & peripheral information processing) in the current study.

Haddock et al. (2008) found that people differ in their general need to have information about matters (need for cognition) and in their need for affect, through praise and reinforcement. They found that a cognitive message is more persuasive among cognition-oriented individuals (i.e., individuals who have a high *need for cognition*), whereas an affective message is more persuasive among affect-oriented individuals (i.e., individuals who have a high *need for affect*). Similar effects were demonstrated by Mayer and Tormala (2010), who suggest that personalizing nutrition information based on peoples’ *need for cognition* and *need for affect* can be effective in stimulating people to make more healthy food choices. Therefore, we included the constructs *need for affect* and *need for cognition* in our study.

Regulatory Focus Theory posits differences between people in motivational orientation, namely a *promotion* or *prevention focus* (Higgins, 1997). People with a *promotion focus*, focus on achieving positive outcomes and primarily think “How would I like to be?”. People with a *prevention focus*, focus on preventing negative outcomes and primarily think “How should I be?”. Research shows that framing information in terms of gains is more effective for people with a *promotion focus*, and using loss-frames is more effective for people with a *prevention focus* (Lee and Aaker, 2004). The majority of studies in the review by Ludolph and Schulz (2015) confirmed that regulatory fit enhances the effectiveness of health messages. Thus, we also included *regulatory focus* in our study.

In the current study the items from the above mentioned psychological constructs have been combined in one online survey (see Table 1; more details on the survey can be found in the “Materials and Methods” section).

**TABLE 1:** Used constructs in the survey, their number of items and the answering scale (and number of answer categories) used (top part); binary questions about the preference for feedback (lower part).

Construct	Number of items	Answering scale (number of answer categories)
Self-regulation	5	Likert (7)
Action self-efficacy	7	Likert (7)
Coping self-efficacy	13	Not difficult – very difficult (7)
Social comparison	10	Likert (7)
Intrinsic motivation	6	Very untrue – true (7)
Health info processing	8 (4 + 4)	Likert (7)
Need for cognition	3	Likert (7)
Need for affect	6 (3 + 3)	Likert (7)
Regulatory focus	10 (5 + 5)	Likert (7)
**Preference for feedback**		
Focus of advice	1	Binary choice
Information activity	1	Binary* choice
Amount of information	1	Binary* choice

*But see Supplementary Appendix 3.

## Materials and Methods

### Sample and Procedure

An online survey was conducted in August 2018 under 1,013 respondents in the Netherlands. The survey was administered by a professional market research company (MSI-ACI Europe BV). The respondents were approached by email. As the data was gathered anonymously and only average scores were used in the analyses, no formal ethical approval needed to be officially obtained. At the start of the study participants were informed about the global goal of the study namely, to map individuals’ choices and opinions about personalized nutrition advice. The participants were explained that they could refrain from filling out the survey at any moment and could quit without providing any reason for their withdrawal. To ensure a nationally representative sample, respondents were quota-sampled based on gender, age, highest level of completed education and income. Due to a lack of variation in their responses, 25 respondents were excluded^1^, thus the final sample consisted of 988 respondents with a completely filled out survey. The final sample contained 486 males and 500 females (2 respondents did not fill out their gender) and a mean age of 46.2 years ranging from 18 to 75 years. These, and some more, demographics can be found in Supplementary Appendix 1.

The questionnaire started with a measurement of the psychological characteristics. Respondents then continued with several multiple choice questions which measured their stated preferences for receiving certain forms of personalized nutrition and health advice or information. Finally, the demographics were assessed.

### Measures

Validated scales were used to measure the various constructs. When necessary, the items were translated to Dutch using back-translation and reversed items were framed positively, because reversed items have shown to be able to lead to measurement problems like unduly complex factor structures (cf. Weijters and Baumgartner, 2012).

*Self-regulation with regard to healthy eating* was measured using a 5-item scale developed and validated by Kliemann et al. (2016). The items were answered on seven-point Likert scales ranging from 1 (strongly disagree) to 7 (strongly agree). *Action self-efficacy with regard to healthy eating* was measured with 7 items (based on Wilson-Barlow et al., 2014). The items were also answered using seven-point Likert scales ranging from 1 (strongly disagree) to 7 (strongly agree). *Coping self-efficacy with regard to healthy eating* was measured with 13 items from the Eating Self-Efficacy Scale (ESES), based on Glynn and Ruderman (1986). These items were answered on seven-point scales ranging from 1 (not difficult at all) to 7 (very difficult). *Social comparison* was measured with a 10-item questionnaire from Gibbons and Buunk (1999). The items were answered on seven-point Likert scales ranging from 1 (strongly disagree) to 7 (strongly agree). *Intrinsic motivation to eat healthily* was measured with 6 items that assessed self-reports of interest and enjoyment of healthy eating (Ryan and Deci, 2000), items were answered on seven-point scales ranging from 1 (very untrue) to 7 (very true). *Healthy information processing* was measured with 8 items −4 items measured central processing and 4 items measured peripheral processing– based on Trumbo and McComas (2003). *Need for cognition* was measured with 3 items based on Cacioppo et al. (1984). *Need for affect* was measured with 3 items that assessed approaching emotions and 3 items that assessed avoiding emotions based on Appel et al. (2012). *Regulatory focus* was measured with 5 items assessing prevention focus and 5 items assessing promotion focus based on Haws et al. (2010). Items for information processing, need for cognition, need for affect and regulatory focus were all measured with seven-point Likert scales ranging from 1 (strongly disagree) to 7 (strongly agree). An overview of the items in the survey is presented in Table 1.

All constructs and their items, means, standard deviations and Cronbach’s alphas are shown in Supplementary Appendix 2. The Cronbach’s alpha’s are all rather high, ranging from 0.71 to 0.95, indicating that our sample answered like expected with respect to the psychological scales, which were designed to show only one underlying concept.

Three questions were asked concerning the preferred way that respondents would like to receive health advice or feedback about their health status (bottom part of Table 1). The following concepts were probed by these questions about receiving personalized feedback on respondents’ health status or receiving personalized health advice (for the verbatim items, see Supplementary Appendix 3):

• Focus of advice: preference for information on either how to obtain positive results, or on how to prevent negative consequences.
• Information activity: preference for looking for advice when the respondent itself wishes it, or to always receive advice on the same fixed moment.
• Amount of information: preference for short, to-the-point information or for detailed information that includes explanations about why the advice is good for the respondent.

For all three items answer categories with low frequencies (below 10%) were deleted from the analyses (see also Supplementary Appendix 3). The 2nd and 3rd answer category from the ‘amount of information’ item were merged. The reason is the fact that the detailed information mentioned in the 2nd category may for many respondents have included what is specifically named in the 3rd. These questions were measured with a single-item, also to keep the survey relatively concise; in many cases, single-item measures perform equally well as multiple-item measures of the same construct (Bergkvist and Rossiter, 2007).

### Analysis of Data

#### Principal Component Analysis

A Principal Component Analysis (PCA) was conducted on the 68 items in the survey (using SPSS v.23), which resulted in a four dimensional solution on which a Varimax rotation was performed. The scores for this four dimensional model are used to predict reported preferences for three different forms in which dietary health information can be provided to people. These predictions have been carried out separately by means of three logistic regression analyses (using SPSS v.23). The reason to carry out three separate logistic regressions, rather than combining these into one model in order to include interactions between dependent variables is rather practical. One of the aims of this study was to explore the possibility to use the psychological profiles obtained to provide tailored dietary advice in a specialized computer program (ultimately in a smartphone-app). The three dependent variables are linked to matters that could be separately implemented in such an app. Furthermore, in hindsight, the correlations between the three variables turned out to be very low (Supplementary Material).

## Results

### Principal Component Analysis

The amount of variance in the four component (correlation) PCA model is 51% and the choice for this four component model is not based on a formal criterion like the eigenvalue > 1 criterion (in a full dimensional PCA, without *post hoc* rotation, this would have yielded a 12 component model with the component 5 through 12 together containing 12% variance). Visual inspection of the scree graph (see Supplementary Material) suggested a four component model, although the fourth component contains a mere 8% of variation. All four components showed an interpretable set of loading items (see Table 2), although the fourth component should be interpreted with caution due to its low amount of explained variance. Loadings with an absolute value < 0.3 are not used to interpret the components nor are they shown in Table 2.

**TABLE 2:** Loadings of items on the four components.

Item from the survey	Component			
	
	1	2	3	4
Action self-efficacy with regard to healthy eating – I am able to eat a variety of healthy foods to keep my diet balanced.	0.749			
Healthy information processing (central) – When the topic of healthy eating comes up, I always try to learn more about it.	0.734			
Intrinsic motivation to eat healthily – I (want to) eat healthily…: because I like being involved with healthy eating.	0.731			
Action self-efficacy with regard to healthy eating – I am able to choose recipes based on nutritional value.	0.731			
Intrinsic motivation to eat healthily – I (want to) eat healthily…: because I enjoy eating healthy.	0.730			
Intrinsic motivation to eat healthily – I (want to) eat healthily…: because I thought about it a lot and I believe it is important for many aspects of my life.	0.721			
Self-regulation with regard to healthy eating – I follow my eating intentions.	0.715			
Action self-efficacy with regard to healthy eating – I am able to modify recipes to make them healthier.	0.710			
Action self-efficacy with regard to healthy eating – Based on my knowledge of nutrition, I am able to choose healthy foods at restaurants and from stores.	0.690			
Self-regulation with regard to healthy eating – I do not get distracted from my eating intentions.	0.672	−0.322		
Intrinsic motivation to eat healthily – I (want to) eat healthily.: because I want to take responsibility for my own health.	0.670			
Self-regulation with regard to healthy eating – If I am not eating in the way I intend to, I make changes.	0.670			
Intrinsic motivation to eat healthily – I (want to) eat healthily…: because I am interested in finding new ways to eat healthy.	0.663			
Action self-efficacy with regard to healthy eating – When I feel hungry, I am able to easily choose healthy food over less healthy options.	0.656			
Healthy information processing (central) – Healthy eating is an important issue, and it has been important to me to decide on how I feel about it.	0.651			
Intrinsic motivation to eat healthily – I (want to) eat healthily…: because it is important to me to be as healthy as possible.	0.650			
Healthy information processing (peripheral) – The information I have at this time meets all of my needs for knowing about how to eat healthy.	0.635			
Healthy information processing (central) – In order to be completely informed about the issue of healthy eating, I feel that the more viewpoints I can get, the better off I will be.	0.635			
Healthy information processing (peripheral) - I feel quite capable of finding and using the information that I need in order to decide how to eat healthy.	0.632			
Healthy information processing (peripheral) – I have been able to make a decision about how concerned I am about not eating healthy by using my existing knowledge.	0.620			
Healthy information processing (central) – I have made a strong effort to carefully examine the scientific information presented on the question of healthy eating.	0.602		0.303	
Action self-efficacy with regard to healthy eating – I am able to consume fruits and vegetables in most of my meals.	0.587			
Action self-efficacy with regard to healthy eating – If I choose to indulge in unhealthy food, I am able to appropriately compensate later.	0.554			
Self-regulation with regard to healthy eating – I’m good at resisting tempting food.	0.507	−0.425		
Healthy information processing (peripheral) – On the issue of healthy eating, I am willing to put my trust in the experts.	0.449			
Self-regulation with regard to healthy eating – I find it easy to remember what I have eaten throughout the day.	0.438			
Coping self-efficacy with regard to healthy eating – It is difficult to keep a healthy diet: when I feel restless.		0.847		
Coping self-efficacy with regard to healthy eating – It is difficult to keep a healthy diet: when I feel upset.		0.836		
Coping self-efficacy with regard to healthy eating – It is difficult to keep a healthy diet: when I feel frustrated.		0.830		
Coping self-efficacy with regard to healthy eating – It is difficult to keep a healthy diet: when I am irritable.		0.827		
Coping self-efficacy with regard to healthy eating – It is difficult to keep a healthy diet: when I am tense.		0.826		
Coping self-efficacy with regard to healthy eating – It is difficult to keep a healthy diet: when I am depressed.		0.826		
Coping self-efficacy with regard to healthy eating – It is difficult to keep a healthy diet: when I am angry.		0.795		
Coping self-efficacy with regard to healthy eating – It is difficult to keep a healthy diet: when tempting food is in front of me.		0.722		
Coping self-efficacy with regard to healthy eating – It is difficult to keep a healthy diet: when I am hungry.		0.692		
Coping self-efficacy with regard to healthy eating – It is difficult to keep a healthy diet: when I am with friends.		0.661		
Coping self-efficacy with regard to healthy eating – It is difficult to keep a healthy diet: during a social occasion dealing with food, like a restaurant or dinner party.		0.627		
Coping self-efficacy with regard to healthy eating – It is difficult to keep a healthy diet: around holiday time.		0.609		
Coping self-efficacy with regard to healthy eating – It is difficult to keep a healthy diet: when I want to enjoy my food.		0.591		
Social comparison – I am the type of person who often compares myself with others.			0.815	
Social comparison – If I want to find out how well I have done something, I compare what I have done with how others have done.			0.801	
Social comparison – I often compare myself with others with respect to what I have accomplished in life.			0.791	
Social comparison – I consider my situation in life relative to that of other people.			0.790	
Social comparison – I often compare how I am doing socially (e.g., social skills and popularity) with other people.			0.786	
Social comparison – I pay a lot of attention to how I do things compared with how others do things.			0.779	
Social comparison – I always like to know what others in a similar situation would do.			0.738	
Social comparison – I often try to find out what others think who face similar problems as I face.			0.686	
Social comparison – If I want to learn more about something, I try to find out what others think about it.			0.651	0.317
Regulatory focus (prevention) – I worry about making mistakes.			0.570	
Need for affect (avoid) – I do not know how to handle my emotions, so I avoid them.			0.562	
Need for affect (avoid) – If I reflect on my past, I see that I tend to be afraid of feeling emotions.			0.526	
Need for affect (avoid) – I find strong emotions overwhelming and therefore try to avoid them.			0.495	
Social comparison – I often like to talk with others about mutual opinions and experiences.			0.467	0.424
Regulatory focus (promotion) – In general I am focussed on reaching positive outcomes.				0.639
Need for cognition – I really enjoy a task that involves coming up with new solutions to problems.				0.623
Need for cognition – I like to have the responsibility of handling a situation that requires a lot of thinking.				0.606
Regulatory focus (promotion) – When I see an opportunity for something I like, I get excited right away.				0.575
Regulatory focus (promotion) – I frequently imagine how I will achieve my hopes and aspirations.			0.314	0.542
Regulatory focus (promotion) – I feel like I have made progress toward being successful in my life.	0.321			0.527
Need for affect (approach) – Emotions help people to get along in life.				0.526
Regulatory focus (promotion) – When it comes to achieving things that are important to me, I find that I perform as well as I would ideally like to.	0.324			0.522
Regulatory focus (prevention) – In general I am focussed on preventing negative outcomes.				0.487
Need for affect (approach) – It is important for me to be in touch with my feelings.				0.462
Need for cognition – I would prefer complex to simple problems.				0.456
Need for affect (approach) – I think that it is important to explore my feelings.				0.430
Regulatory focus (prevention) – I frequently think about how I can prevent failures in my life.			0.325	0.426
Regulatory focus (prevention) – In general I obey rules and regulations.				0.376
Regulatory focus (prevention) – Being careful has prevented me from getting into trouble at times.				0.300

**Only loadings with an absolute value ł 0*.*3 are shown*.*

The first component (18% VAF) shows items that concern an intrinsic interest in healthy eating, combined with the will (and capability) to maintain a healthy diet. The first component (18% VAF), coined ‘*intrinsic interest and capabilities for healthy eating*,’ combines six items from the scale ‘intrinsic motivation for healthy eating,’ seven items from ‘action self-efficacy concerning healthy eating,’ eight items from ‘information processing concerning healthy eating,’ and five items from ‘self-regulation concerning healthy eating.’ These items have loadings over 0.44 on this component.

The second component (13% VAF) contains all items regarding ‘coping self-efficacy’ (Schwarzer and Renner, 2000). These items all concern a difficulty in maintaining a healthy diet, under a broad range of circumstances, which includes a variety of eating occasions and emotional occurrences. These items can therefore be viewed as (a general lack of) coping self-efficacy to eat healthily. We label this second component “*perceived difficulty to eat healthily*.”

The third component (12% VAF) shows a general attitude which we interpret as a form of ‘*self-worth insecurity*.’ It contains the ten items from the ‘social comparison’ scale, three ‘emotion avoidance’ items from the ‘need for affect’ scale and one ‘prevention focus’-item from the ‘regulatory focus’ scale. Most items loading on this component (loadings over 0.47) point to a social comparison orientation and an avoidance to experience emotions.

The fourth component contains a mere 8% of variance and contains the three items from the ‘need for cognition’ scale, the three ‘approach emotions’-items from the ‘need for affect’ scale, five ‘promotion focus’-items and one ‘prevention focus’-item from the ‘regulatory focus’ scale. These items have loadings over 0.43 on this component. Thus, the items in this component appear related to seek cognitive challenges, seek positive results and seek the experience of emotions. We interpret and label this component as “*seeking positive challenges*.” Three other ‘prevention focus’-items also loaded on this component, however, we decided not to take this into consideration because they either had low loadings (below 0.38) or a similar (low) loading on more than one component.

In summary, we have thus reified the four components as follows:

(1) Intrinsic interest and capabilities for healthy eating,
(2) Perceived difficulty to eat healthily,
(3) Self-worth insecurity,
(4) Seeking positive challenges.

### Relation Between the Components and Personalized Health Feedback Preferences

The three separate logistic regressions show that some of the four principal components predict some of the three dependent variables. The results of the three logistic regressions are given in Table 3. Significant (*p* < 0.05) results are indicated by italicizing the corresponding lines in the table.

**TABLE 3:** Results of the three logistic regressions, Lines holding significant predictions (p < 0.05) italicized [in the table: regression weight b, their standard errors (s.e.), Wald statistics, significance values, odd ratios and the lower and upper borders of the 95% confidence interval for the odd ratios, respectively].

Focus of advice [χ^2^(4) = 12.7, *p* = 0.013]	*b*	*s.e.*	Wald	Sig.	Odd ratio	95% c.i. for odd ratio	
	
						Lower	Upper
Component 1	−0.139	0.094	2.156	0.142	0.870	0.723	1.048
Component 2	−0.140	0.096	2.131	0.144	0.869	0.720	1.049
Component 3	0.170	0.098	3.014	0.083	1.185	0.978	1.436
*Component 4*	−*0.223*	*0.096*	*5.408*	*0.020*	*0.800*	*0.663*	*0.966*
Constant	−1.94	0.098	391.357	0.000	0.144		
**Information activity** [χ^2^(4) = 18.9, *p* = 0.001]							
Component 1	0.017	0.080	0.045	0.832	1.017	0.869	1.191
*Component 2*	*0.164*	*0.081*	*4.111*	*0.043*	*1.178*	*1.005*	*1.380*
*Component 3*	*0.302*	*0.080*	*14.240*	*0.000*	*1.353*	*1.156*	*1.582*
Component 4	0.032	0.080	0.164	0.685	1.033	0.883	1.209
Constant	−1.07	0.078	186.478	0.000	0.342		
**Amount of information** [χ^2^(4) = 32.7, *p* < 0.000]							
*Component 1*	*0.196*	*0.069*	*8.070*	*0.005*	*1.217*	*1.063*	*1.394*
Component 2	0.102	0.068	2.233	0.135	1.107	0.969	1.266
*Component 3*	*0.323*	*0.069*	*22.027*	*0.000*	*1.381*	*1.207*	*1.581*
Component 4	−0.014	0.068	0.040	0.841	0.986	0.863	1.128
Constant	0.064	0.068	0.889	0.346	1.066		

**In parentheses below the dependent variables name the model χ^2^ its d.f.’s and significance value p*.*

**FIGURE 1:**
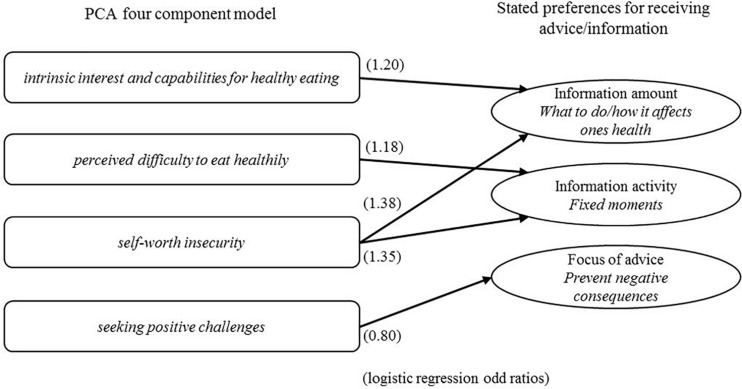
Overview of the significant components (*p* > 0.05), in three separate logistic regressions, (with odd ratios) predicting preferences for receiving health advice/information.

Figure 1 presents an overview of the findings, the extent to which the four components can predict the stated preferences for receiving advice/information.

The logistic regression predicting *Focus of advice* (a preference to receiving advice pointing at ways to prevent negative consequences) shows a significant effect (*p* = 0.02) only for the 4th component (‘*seeking positive challenges*’), with an odd ratio of 0.80. An increase of the score on the 4th component results in a lowering of the probability of preferring to receive advice when it shows how to prevent negative consequences.

*Information activity* (a preference to receiving information on fixed moments) shows to be predicted by both the 2nd component (*perceived difficulty to eat healthily*, *p* = 0.04) and the 3rd component (*self-worth insecurity*, *p* = 0.00). A higher score on the 2nd component results in a heightened (odd ratio 1.18) probability for a preference for receiving information on a fixed moment, analogously does a higher score on component 3 with an odd ratio of 1.35.

The third logistic regression shows that *amount of information* (a preference to receiving detailed information pointing out what to do and how this affects one’s health) can be predicted by the 1st (*p* = 0.01) and 3rd (*p* = 0.00) component (‘*intrinsic interest and capabilities for healthy eating’* and ‘*self-worth insecurity,’* resp.). A higher score on component 1 results in a 1.22 times higher probability of preferring detailed information, a higher score on component 3 in a 1.38 times higher probability of preferring detailed information.

## Discussion

Personalizing nutrition information can be an effective manner to increase compliance and lead to consumers making more healthy food choices (e.g., Brug et al., 2003; Elder et al., 2009). However, previous studies have mostly used a biological/medical basis to personalize nutrition information. In the current study, we extend previous work that shows a relation between psychological characteristics and both dietary choices (e.g., Mayer and Tormala, 2010; Ludolph and Schulz, 2015; Macready et al., 2018) and compliance with personalized advice (Berezowska et al., 2015; Livingstone et al., 2016; Celis-Morales et al., 2017; Rankin et al., 2017; Reinders et al., 2020) and aim to develop a psychological approach to personalize nutrition information based on an elaborate questionnaire (Macready et al., 2018). Furthermore, the current study also explores the relation between the (psychological) components and respondent preferences for certain forms in which dietary information can be provided. The latter provides an indication of the degree to which this psychological approach to personalize nutrition information can be a basis to predict respondent preferences for how respondents would like to receive nutrition information (e.g., in detailed form or not). Ultimately the findings can be used for the development of smartphone apps by which consumers can choose to receive dietary advice in a manner befitting their psychological profile.

### Principal Components

In the current study, we conducted a PCA which delivered four components: *intrinsic interest and capabilities for healthy eating*, *perceived difficulty to eat healthily*, *self-worth insecurity* and *seeking positive challenges*.

The first component (‘intrinsic interest and capabilities for healthy eating’) combines all the items from the action self-efficacy, self-regulation, intrinsic motivation and information processing scales. That the four scales jointly load on the first component in this study, hence correlate, can mean that they measure the same underlying construct in the context of food and healthy eating. Scores on this component increase as respondents have a higher intrinsic motivation and a higher involvement in eating healthily (this is the central route in the ELM of Cacioppo et al., 1984), a higher action self-efficacy and a higher self-regulation. We interpret this as the higher people score on this component, the more they show a combination of an intrinsic interest and having the capabilities to eat healthily. Intrinsic motivation and a high involvement in a certain issue pertain to an intrinsic interest, in this case for healthy eating. Action self-efficacy and self-regulation pertain to having the capability to eat healthily, according to respondents’ own perception. Self-efficacy has also been seen as a motivational aspect of self-regulation (Dinsmore et al., 2008), which theoretically connects action self-efficacy to both self-regulation and intrinsic motivation.

Motivation and capability are often treated as separate concepts in behavioral theories (e.g., Social Cognitive Theory, Bandura, 1977; Health Action Process Approach model, Schwarzer, 1992, 1999; Theory of Planned Behaviour Ajzen and Manstead, 2007). However, the two concepts are important in the sense that behavioral models such as the Motivation, Opportunity, Ability (MOA) model (Ölander and Thøgersen, 1995) state, that behavior change is more likely when an individual is both motivated and capable to change one’s behavior.

Based on this first PCA-component, it can be suggested that the motivational aspect of self-regulation, is indeed tied to respondents’ level of self-efficacy to change their diet. Self-regulation also has a more cognitive part, which in this component is reflected in the form of information processing. Previous work has linked information processing with self-regulation, specifically with regard to self-regulated learning (e.g., Winne, 2001). This can be interpreted for the current study, as respondents aiming to learn how to eat more healthily in a self-regulated manner, and processing the necessary information in order to do so.

Items referring to difficulties with following a healthy diet load together on the second component (‘perceived difficulty to eat healthily’). These items all stem from the same self-efficacy scale (Glynn and Ruderman, 1986). That they score uniquely on this component, and apparently do not clearly correlate with any other items in this survey shows that they act as a single force in the whole of the survey in this study. There thus seems to be a general trend which is strong enough to surface in our sample of 988 respondents. This is not in line with the original scale (Glynn and Ruderman, 1986), which comprises of two components, namely difficulty eating healthily when experiencing negative affect (NA) and difficulty eating healthily during socially acceptable circumstances (SAC). In the specific food and health context of this survey these two components may not have been distinguished by the respondents, in the sense that difficulty to eat healthily in terms of NA and SAC appears to be connected to each other. This may indicate that the component structure can depend on the specific context of this survey.

The fact that the first and second component are built from domain-specific items explicitly concerning food and/or health is noteworthy. This may point at a methodological artifact where items that are comparably stated, or are on similar matters, are scored alike. The reason that the first and second component are separate components, can be explained by the fact that the components have a different focus (Bandura, 1977). Items in the second component focus on the *difficulty* to perform healthy dietary behaviors, whereas the items in the first component focus on *positive motivations and capabilities* with regard to eating healthily. And as mentioned before, reversed items often result in a separate component (Weijters and Baumgartner, 2012).

Most items loading on the third component (‘self-worth insecurity’) point to psychosocial characteristics of a type of individual that compares his/her behavior to others in order to judge his/her own behavior, and at an avoidance to experience emotions. One reason why people might structurally compare themselves to others is that they are insecure about themselves (Maslow et al., 1945; White et al., 2006) and have a lower self-worth (Crocker and Wolfe, 2001). Similarly, people attempt to avoid emotions when emotional lows are expected that affect one’s self-worth (Crocker and Knight, 2005), thus avoiding emotions can be tied to maintaining one’s self-worth.

The fourth component (‘seeking positive challenges’) combines items from the ‘need for cognition’ scale, the ‘approach emotions’-items from the ‘need for affect’ scale, and ‘promotion focus’-items and ‘prevention focus’-item from the ‘regulatory focus’ scale. The latter two sets of items are not easy to reconcile as this component seems to combine a promotion focus with a prevention focus, which are traits believed to be not in line with each other. We must again stress that the fourth component represents a rather low amount of variance in the data, so it is does not represent a very strong force to explain this result. Nevertheless it appeared interpretable as ‘seeking positive challenges’, and we have included it in the prediction models.

We observe that the first two components are food related components. Component 3 and 4 are related to general psychosocial characteristics, which are not specifically related to food. This suggests that, in these results, answers to the food related items are unrelated to answers to general items, as the four components are uncorrelated (the varimax rotation maintains orthogonality of the PCA components).

### Prediction of Feedback Preferences

This first component (‘intrinsic interest and capabilities for healthy eating’) shows up as predictor of the ‘information amount’ in the logistic regression. A mind set for maintaining a healthy diet seems to go together with an interest in receiving an advice/information on what do to, together with information on how the advice will affect one’s health, compared to only receiving an advice. So individuals who have intrinsic interest and capabilities to eat healthily prefer extra information on why an advice is good for their health in addition to receiving dietary advice.

The second component (‘perceived difficulty to eat healthily’), tallies with it predicting a preference for a fixed moment to receive information/advice. This may be a strategy of those that perceive a difficulty in themselves, to help them control their healthy food intake. They may see themselves unable to cope with a freedom to find information on their own account, hence making it more likely that a form of advice is chosen that is received on a fixed moment.

Component 3 (‘self-worth insecurity’), together with the first component, predicts a preference to receiving information/advice together with instructions about what to do and how it relates to one’s health. Component 3, together with the second component, also predicts a preference for a fixed moment to receive information/advice. The insecurity of the individuals scoring high on this component seems to lead to a wish for specific information/advice about their health situation at fixed moments in time. The social comparison part of the third component, seems to be in line with wanting to receive specific information and instruction with the advice, as the advice comes from others, which enables a social comparison.

The fourth component negatively predicts the preference for advice with a focus on preventing negative consequences. As this component models ‘seeking positive challenges’, this seems to make sense, with the proviso that the merger of both a prevention and a promotion focus appears strange. Note that this component contains a mere 8% variance in the data, so this component’s size is not very large, which may lead to the possibility that it combines small effects, despite their being theoretically not compatible.

That four scales, adapted to focus on food and health, together make up the first component suggests that these four underlying constructs merge when seen in the context of food and health. Intrinsic motivation, self-efficacy, information processing and self-regulation appear to correlate highly when seen in the context of food and health. This points at the possibility to reduce the set of items in future surveys designed to probe relevant psychological parameters of a sample of respondents, in a food and health context.

### Study Limitations

One potential limitation of the study is that for the ‘preference for feedback’ constructs single-item measures were used. However, in many cases single-item measures perform just as well as multiple-item measures (Bergkvist and Rossiter, 2007). Since the psychological constructs are relatively complex, we decided to use validated, multiple-item scales for those constructs, but to use single-item measures for the more simple choice constructs. Also keeping in mind the need to keep the survey relatively concise. Future research could test whether the findings of the current study are similar when also using multiple-item measures for the choice constructs.

Furthermore, though we aimed to keep the survey concise, potentially part of the respondents could have experienced a sense of respondent fatigue. Typically, an average maximum length of 20 min is recommended for a survey before respondent fatigue becomes an issue (Cape and Phillips, 2015; Revilla and Ochoa, 2017). This was taken into account when designing the survey, also by limiting the number of items. It was also agreed with the market research company before the survey was sent out to double-check the survey would take no longer than 20 min to complete.

For the current study, an online survey was administered relying on self-reports from participants. This can have certain disadvantages as is discussed by McDonald (2008), such as for instance participants responding in manners through which they can view themselves as favorable. To account for these potential drawbacks of self-reports, follow-up research can include actual behavioral measures with actual consequences, such as performing a study where the outcome variable is not a self-reported choice, but actually choosing a certain format of nutrition advice that participants subsequently receive.

## Conclusion

This study shows that it is possible to use psychological characteristics to predict the way consumers would like to receive advice/information about their health status (and diet). It also points out that there are different personalities of consumers, that may benefit from being addressed according to their preference for receiving advice/information on specific moments, of a specific level of detail and pointing at the type of consequences the advice has. When developing a computer program (typically a smartphone app) it is advisable to tailor the way health advice is provided to the type of consumer. The latter can be assessed by, e.g., asking the user of the app some questions upon installation of the app. The long survey in this study is not suited for this, but the PCA points at a way to reduce the length to a low number of questions.

Compliance to an advice was not addressed in this study, obviously that is the ultimate ‘proof of the pudding’ that needs to be further investigated, possibly with the survey presented here as an instrument for tailoring advice. Based on the four component PCA model a reduction of items can be achieved, to in a more concise manner personalize nutrition advice. Of course this basically means the construction of a new psychological scale, specifically focused at ‘food and health’-related psychological characteristics. Such a scale will need to be studied and analyzed in detail before it can validly be applied in subsequent studies in this field.

## Data Availability Statement

The raw data supporting the conclusions of this article will be made available by the authors, without undue reservation.

## Ethics Statement

Ethical review and approval was not required for the study on human participants in accordance with the local legislation and institutional requirements. Written informed consent for participation was not required for this study in accordance with the national legislation and the institutional requirements.

## Author Contributions

All authors listed have made a substantial, direct and intellectual contribution to the work, and approved it for publication.

## Conflict of Interest

The authors declare that the research was conducted in the absence of any commercial or financial relationships that could be construed as a potential conflict of interest.

## Footnotes

1 These 25 respondents had 0 standard deviation in their responses to items measuring regulatory focus (prevention focus vs. promotion focus) and/or need for affect (approach vs. avoidance of affect), in combination with scoring only ‘1,’ ‘2,’ ‘6,’ or ‘7’ in all these responses. Given that these two constructs contain opposite items (e.g., A ‘7’ in one item indicates a high prevention focus, while a ‘7’ in a next item indicates a high promotion focus), these 25 respondents were excluded.
