# Supplementary material for: Personalized Nutrition Advice: Preferred Ways of Receiving Information Related to Psychological Characteristics
Source: Front Psychol. 2021 Jun 22;12:575465. doi: 10.3389/fpsyg.2021.575465 (PMC8258260; doi:10.3389/fpsyg.2021.575465)
Supplement: Supplementary file 1 [file Data_Sheet_1.docx]

Appendix 1 Table with sample demographics.

|  | % |
| --- | --- |
| Gender  Male  Female  Not mentioned | 49.2  50.6  0.2 |
| Age (*M*=46.2; range=18-75)  18 – 24 years  25 – 34 years  35 – 49 years  50 – 64 years  > 64 years | 11.7  16.8  27.8  28.8  14.8 |
| Education  Low  Medium  High | 18.9  45.7  35.3 |
| Net monthly household income  < €1.500  €1.500 - €3.000  €3.000 - €7.500  > €7.500  I do not know / will not say | 14.4  38.6  26  2.2  18.8 |
| Health problem (multiple answers)  No  Overweight  Diabetes  High blood pressure  Gastrointestinal diseases  High cholesterol  Cardiovascular diseases  Food allergies  Lung diseases  Osteoporosis  Other | 53.8  24  6  10.7  7.5  8.9  3.8  5.5  4.3  3  4.8 |
| Office job  Yes  No | 33.2  66.8 |
| Number of people in household  1 person  2 people  3 people  4 people  5 people  6 people or more | 25.7  38.9  13.4  15.3  5  1.8 |

Appendix 2 Constructs, items, means (M), standard deviations (Sd) and Cronbach’s alpha (α) for each individual scale.

| Constructs and items | M | Sd | α |
| --- | --- | --- | --- |
| **Self-regulation with regard to healthy eating**  1. I’m good at resisting tempting food.  2. I follow my eating intentions.  3. I do not get distracted from my eating intentions.  4. I find it easy to remember what I have eaten throughout the day.  5. If I am not eating in the way I intend to, I make changes. | 4.76 | 1.15 | .86 |
| **Action self-efficacy with regard to healthy eating**  1. I am able to consume fruits and vegetables in most of my meals.  2. I am able to eat a variety of healthy foods to keep my diet balanced.  3. Based on my knowledge of nutrition, I am able to choose healthy foods at restaurants and from stores.  4. I am able to modify recipes to make them healthier.  5. I am able to choose recipes based on nutritional value.  6. If I choose to indulge in unhealthy food, I am able to appropriately compensate later.  7. When I feel hungry, I am able to easily choose healthy food over less healthy options. | 5.02 | 1.06 | .90 |
| **Coping self-efficacy with regard to healthy eating**  It is difficult to keep a healthy diet...  1. when I feel restless.  2. around holiday time.  3. when I feel upset.  4. when I am tense.  5. when I am with friends.  6. when I am irritable.  7. during a social occasion dealing with food, like a restaurant or dinner party.  8. when I am angry.  9. when I am depressed.  10. when I want to enjoy my food.  11. when I feel frustrated.  12. when tempting food is in front of me.  13. when I am hungry. | 4.08 | 1.37 | .95 |
| **Social comparison**  1. I pay a lot of attention to how I do things compared with how others do things.  2. If I want to find out how well I have done something, I compare what I have done with how others have done.  3. I often compare how I am doing socially (e.g., social skills, popularity) with other people.  4. I am the type of person who often compares myself with others.  5. I often compare myself with others with respect to what I have accomplished in life.  6. I often like to talk with others about mutual opinions and experiences.  7. I often try to find out what others think who face similar problems as I face.  8. I always like to know what others in a similar situation would do.  9. If I want to learn more about something, I try to find out what others think about it.  10. I consider my situation in life relative to that of other people. | 3.80 | 1.32 | .94 |
| **Intrinsic motivation to eat healthily**  I (want to) eat healthily...  1. because I want to take responsibility for my own health.  2. because I thought about it a lot and I believe it is important for many aspects of my life.  3. because it is important to me to be as healthy as possible.  4. because I like being involved with healthy eating.  5. because I am interested in finding new ways to eat healthy.  6. because I enjoy eating healthy. | 5.24 | 1.10 | .90 |
| **Healthy information processing**  *Central processing*  1. In order to be completely informed about the issue of healthy eating, I feel that the more viewpoints I can get, the better off I will be.  2. I have made a strong effort to carefully examine the scientific information presented on the question of healthy eating.  3. When the topic of healthy eating comes up, I always try to learn more about it.  4. Healthy eating is an important issue, and it has been important to me to decide on how I feel about it.  *Peripheral processing*  5. On the issue of healthy eating, I am willing to put my trust in the experts.  6. The information I have at this time meets all of my needs for knowing about how to eat healthy.  7. I feel quite capable of finding and using the information that I need in order to decide how to eat healthy.  8. I have been able to make a decision about how concerned I am about not eating healthy by using my existing knowledge. | *4.55*  *5.16* | *1.26*  *1.11* | *.88*  *.84* |
| **Need for cognition**  1. I would prefer complex to simple problems.  2. I like to have the responsibility of handling a situation that requires a lot of thinking.  3. I really enjoy a task that involves coming up with new solutions to problems. | 4.51 | 1.28 | .83 |
| **Need for affect**  *Approach emotions*  1. It is important for me to be in touch with my feelings.  2. I think that it is important to explore my feelings.  3. Emotions help people to get along in life.  *Avoid emotions*  4. I do not know how to handle my emotions, so I avoid them.  5. I find strong emotions overwhelming and therefore try to avoid them.  6. If I reflect on my past, I see that I tend to be afraid of feeling emotions. | *4.77*  *3.59* | *1.13*  *1.47* | *.81*  *.85* |
| **Regulatory focus**  *Promotion focus*  1. When it comes to achieving things that are important to me, I find that I perform as well as I would ideally like to.  2. I feel like I have made progress toward being successful in my life.  3. When I see an opportunity for something I like, I get excited right away.  4. I frequently imagine how I will achieve my hopes and aspirations.  5. In general I am focussed on reaching positive outcomes.  *Prevention focus*  6. In general I obey rules and regulations.  7. Being careful has prevented me from getting into trouble at times.  8. I worry about making mistakes.  9. I frequently think about how I can prevent failures in my life.  10. In general I am focussed on preventing negative outcomes. | *4.81*  *4.70* | *0.92*  *0.92* | *.79*  *.71* |

Appendix 3 Items asking preferences for receiving personalised feedback on respondents’ health status or receiving personalised health advice (translated from Dutch), deleted answer categories (less than 10% respondents), merged categories, and between parentheses the percentage of respondents selecting the answer categories.

**Focus of advice**

If you could obtain nutritional advice specifically aimed at your situation, which focus would have your preference?

1. Information on how you can obtain positive result (*e.g.* ‘Choose soup with your bread meal, in order to lose weight.’). (87)

2. Information on how you can prevent negative consequences (*e.g.* ‘Do not choose snacks, so you do not gain weight.’). (13)

**Information activity**

If you could obtain nutritional advice specifically aimed at your situation, in what way would you preferably receive this information?

1. I want to be able to access the information when it suits me. (66)

2. I like to receive alerts on a fixed time (that I can set myself) of the day. (23)

*Deleted answer categories:*

- I’d like to receive messages automatically, always when I am in a specific location, *e.g.* in the supermarket or at a party. (7)
- Other (4)

**Amount of information**

If you could obtain nutritional advice specifically aimed at your situation, how much explanation would you wish?

1. I would like to have a short and clear message about what I should do. (45)

2. I want to know what to do, including detailed information. (23)

3. I want to know what to do and why this is important for my current health. (24)

*Deleted answer category:*

I want to know what to do to prevent future health problems. (8)
