# Supplementary material for: Personalized Nutrition Advice: Preferred Ways of Receiving Information Related to Psychological Characteristics
Source: Front Psychol. 2021 Jun 22;12:575465. doi: 10.3389/fpsyg.2021.575465 (PMC8258260; doi:10.3389/fpsyg.2021.575465)
Supplement: Supplementary file 2 [file Table_1.DOCX]

**Supplementary material**

with

Personalised nutrition advice: preferred ways of receiving information related to psychological characteristics. (G.B. Dijksterhuis, E. Bouwman, D. Taufik)

Scree graph of the PCA.


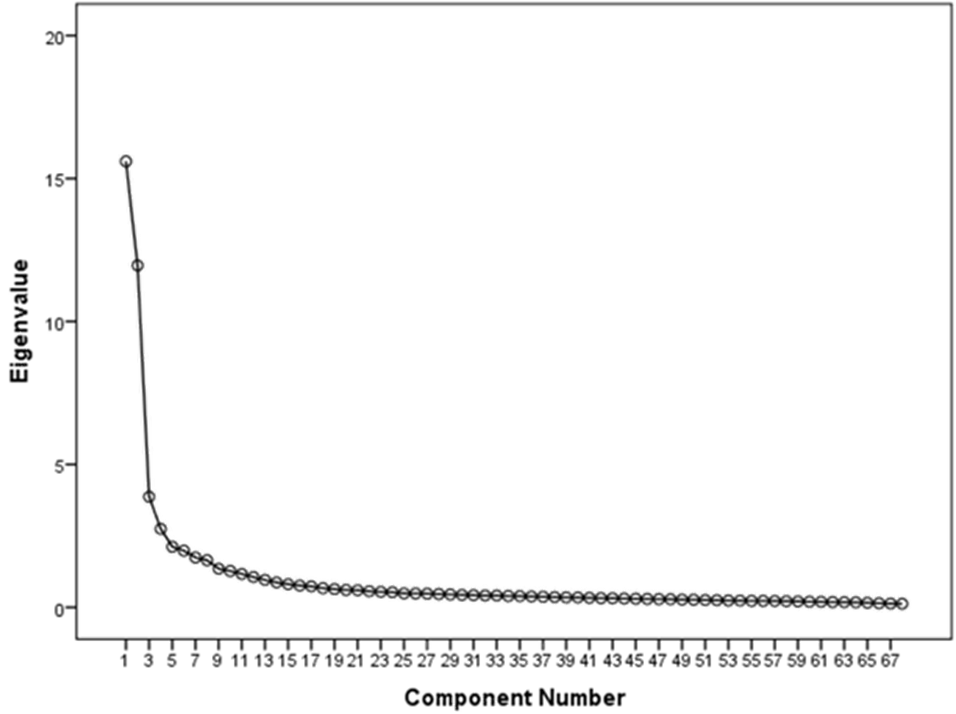


Bivariate Pearson correlations between the three dependent variables.

|  | Information activity | Amount of information |
| --- | --- | --- |
| Focus of advice | 0.037 | -0.012 |
| Information activity |  | -0.068* |

** p* = 0.042
